# Specific Correlation between the Hegu Point (LI4) and the Orofacial Part: Evidence from an fMRI Study

**DOI:** 10.1155/2015/585493

**Published:** 2015-09-13

**Authors:** Su-ping Kong, Qi-wen Tan, Ying Liu, Xiang-hong Jing, Bing Zhu, Yong-jun Huo, Bin-bin Nie, Dian-hui Yang

**Affiliations:** ^1^Institute of Acupuncture and Moxibustion, China Academy of Chinese Medical Sciences, Beijing 100700, China; ^2^Department of Acupuncture and Moxibustion, The Affiliated Hospital of Shandong University of Traditional Chinese Medicine, Jinan 250011, China; ^3^Department of Acupuncture and Moxibustion, Shandong University of Traditional Chinese Medicine, Jinan 250014, China; ^4^Department of Radiology, The Affiliated Hospital of Shandong University of Traditional Chinese Medicine, Jinan 250011, China; ^5^Division of Nuclear Technology and Applications, Institute of High Energy Physics, Chinese Academy of Sciences, Beijing 100049, China; ^6^Beijing Engineering Research Center of Radiographic Techniques and Equipment, Beijing 100049, China

## Abstract

Acupoint specificity is a foundational concept in acupuncture theory. It is closely related to the function of the acupoint. In this study, we sought to probe the central mechanisms of the specific correlation between LI4 and orofacial part in Bell's palsy patients. In total, 36 patients with left Bell's palsy were divided into three groups in random order, and each group received transcutaneous electrical acupoint stimulation (TEAS) at only one of three acupoints (LI4, ST6, and a sham point). A single-block fMRI design paradigm was applied to separately detect neural activity related to different stages of TEAS (prestimulation resting state, stimulation, and poststimulation resting state). Functional magnetic resonance imaging data were acquired during TEAS. There were extensive neuronal activities in the LI4 and ST6 groups and significant differences between stimulation at real and sham points. Brain regions were activated more by real acupoint TEAS than by sham point TEAS. Brain regions that were activated with LI4 and ST6 were broadly overlapping and adjacent. Our results provide supplementary neuroimaging evidence for the existence of acupoint specificity. These results may confirm the central mechanisms of the specific correlation between the Hegu point and the orofacial part.

## 1. Introduction

Acupuncture is an ancient method of treating disease that has been in use for more than 2000 years. In recent years, it has gained popularity as an alternative and complementary therapeutic intervention in Western medicine. According to the traditional theory of acupuncture, stimulation at specific acupoints can be used to treat certain diseases.

Bell's palsy is an acute, unilateral idiopathic facial nerve (CN VII) paralysis of unknown etiology. Acupuncture is a common method in the treatment of Bell's palsy in China; the curative effect is significant [1, 2]. However, two studies concluded that there is inadequate evidence to support the effectiveness of acupuncture for Bell's palsy [3, 4].

Transcutaneous electrical acupoint stimulation (TEAS) is an electrical stimulation method which is to use skin electrodes to input specific low frequency pulse into the acupoints to treat diseases. It overcomes the drawbacks, such as discomfort or pain, performed as a form of noninvasive electrical stimulation. It is more acceptable than traditional acupuncture by patients [5]. However, the physiological mechanisms are still unclear [6]. The mode of stimulation or location of acupuncture points is specific to producing different physiological effects [7].

Neuroimaging techniques have provided new insights into the anatomy and physiological function underlying acupuncture [8–12]. In the last 20 years, extensive functional magnetic resonance imaging (fMRI) studies have been conducted to investigate the neurophysiologic mechanism of acupuncture. It is generally agreed that the brain and nervous system play a leading role in processing acupuncture stimuli [13, 14].

Many clinical studies have suggested a remediation role of acupuncture at the Hegu acupoint for Bell's palsy. The Hegu acupoint, also known as “Large intestine 4” (LI4), is the yuan point of the large intestine channel of hand-yangming, at the midpoint on the radial side of the second metacarpal. The large intestine channel passes through the cheek. It crosses the opposite channel at the philtrum. From there, the left channel goes to the right and the right channel to the left, to the contralateral sides of the nose. Thus, the acupuncturists usually choose the contralateral Hegu point to treat Bell's palsy according to the running characteristics of channel of hand-yangming in facial area. The Jiache acupoint, known internationally as “Stomach 6” (ST6), is the point of the stomach channel of foot-yangming; at the belly of the masseter muscle with teeth clenched, one finger width anterior and superior to the angle of the mandible is used frequently for Bell's palsy.

Thus, a study on the brain response to TEAS in Bell's palsy may be helpful in explaining the mechanisms of acupuncture. Here, we chose to evaluate whether there was overlap and adjacent brain activation in fMRI based on LI4 and ST6, which has rarely been reported. The response of the brain to acupuncture is dependent on its functional status [15]. In the present study, we used fMRI to gain insight into the role of TEAS at LI4 and ST6 in Bell's palsy. Thus, the primary purpose of this study was to explore the possible central mechanism underlying the specific correlation between LI4 and the orofacial part in patients with Bell's palsy.

## 2. Methods

### 2.1. Subjects

In total, 36 patients with left Bell's palsy (14 females, mean age, 40.7 ± 1.7 years) were included in this study after providing informed consent. The patients were recruited at the Acupuncture and Moxibustion Department of the Affiliated Hospital of Shandong University of Traditional Chinese Medicine.

They were diagnosed using criteria for Bell's palsy and were selected by means of the following criteria: (1) right handed, according to the modified Edinburgh Handedness Questionnaire of Oldfield [16], (2) between 18 and 60 years of age, (3) history within 1 month, (4) no central nervous system disease, (5) no mental disease, and (6) no other serious diseases.

This study was approved by the Medical Research Ethics Committee and Institutional Review Board of the Affiliated Hospital of Shandong University of Traditional Chinese Medicine.

### 2.2. Experimental Protocol

**Figure 1:**
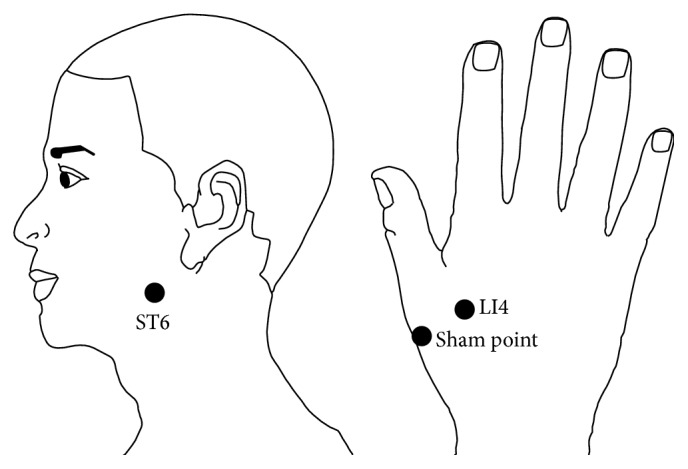
Localization of stimulated acupoints.

In accordance with the project's scheme, the patients were divided randomly into three study groups: 12 cases in the contralateral Hegu stimulus group, 12 cases in the contralateral sham point group (after the first digit, LI4 point by 2 cm), and 12 cases in the ipsilateral Jiache point group (the localization of stimulated acupoints; see Figure 1). In the ipsilateral stimulus group, the stimulus was performed on the same side as that of facial palsy during acquisition of acupuncture fMRI data.

The skin electrodes were placed on the selected acupoints and the ipsilateral elbow point. The HANS Acupuncture Point Nerve Stimulator (HANS-200A, Nanjing, China) was connected with the skin electrode using a home-made extended electromagnetic shielding line. The electrode is self-adhesive. Its conductive body is made of carbon fiber. It does not contain metal. The wires in the electromagnetic shielding line are made of copper. The stimulation parameters were as follows: consistent pulse current output (pulse width: 0.2 ms), electric frequency 5 Hz, and electric current 3 mA. At the end of the experiment, the subjects completed questionnaires about the sensations experienced at the stimulated acupoints.

### 2.3. Data Acquisition

**Figure 2:**
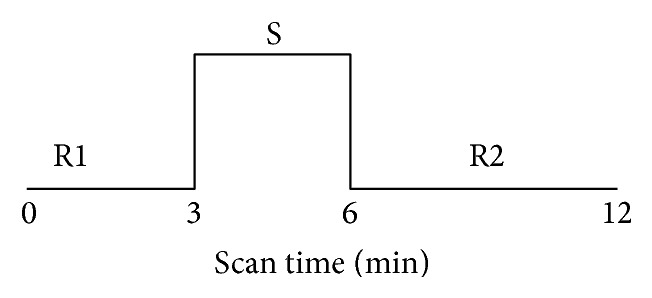
Experimental paradigm.

Magnetic resonance imaging data were acquired using a 3.0-Tesla Signa (Philips) MR scanner. In this study, we adopted a single-block design [17] with 3 min of continuous electric stimulation. For a baseline control, a resting state (REST) scan was conducted for 3 min with no stimulation. Then, the electroacupuncture apparatus was connected for 3 min. Next, the power was turned off and another resting state scan was conducted for 6 min with no stimulation (see Figure 2). During the scanning, the participant was supine on the scanner bed, wearing earplugs to suppress scanner noise, and with the head immobilized by cushioned supports. Subjects were asked to keep their eyes closed and remain relaxed without engaging in any mental task. According to participants' reports after the scanning, they confirmed being awake during the whole process.

T1-weighted images (TR = 8 ms, TE = 3.7 ms, flip angle = 8°, FOV = 250 × 250 mm, matrix = 256 × 256, slice thickness 0.6 mm, gap 0 mm) were obtained to show the anatomy. T2-weighted images were used to assess whether there was any obvious disease of the brain. Whole-brain functional data were acquired using a gradient echo-planar imaging (EPI) sequence (TR = 3000 ms, TE = 35 ms, flip angle = 90°, FOV 230 × 230 mm, matrix = 128 × 128, slice thickness 5 mm, gap 1 mm).

### 2.4. Data Processing and Statistical Analysis

All functional image after processing was performed by a single experienced observer, who was unaware to which patients the scans belonged. The preprocessing and data analysis were performed using the statistical parametric mapping (SPM8) software (Wellcome Department of Imaging Science; http://www.fil.ion.ucl.ac.uk/spm).

The functional data sets of all individuals were preprocessed (corrected for slice acquisition times, corrected for head motion, spatially standardized into the MNI space, and smoothed with a Gaussian kernel of 8 mm full width at Half-maximum). For each smoothed individual image, a fixed-effects analysis was performed based on the general linear model with a box-car response function as the reference waveform, convolved with the canonical hemodynamic response function. The cerebral areas activated during acupuncture at the real acupoint and the nonacupoint, relative to baseline, were obtained. At the second level, to acquire the specific active areas induced by stimulating at the Hegu acupoint, compared with the Jiache acupoint, a group analysis was performed by random-effects analysis based on the two-sample *t*-test model with the results of the first level (height threshold, *P* < 0.005, uncorrected, spatial extent threshold, cluster >20 voxels). The coordinates in Talairach space were obtained by applying the Matthew Brett correction (mni2tal: http://imaging.mrc-cbu.cam.ac.uk/imaging/MniTalairach) to the SPM-MNI coordinates.

## 3. Results

### 3.1. Results of TEAS in the LI4 Group

Compared with the resting state, TEAS at the LI4 acupoint activated brain regions primarily in the left cerebellum, left superior temporal gyrus (BA22), left inferior temporal gyrus (BA20), left inferior parietal lobule (BA5), left middle frontal gyrus (BA8), left postcentral gyrus (BA1/2), and right precentral gyrus (BA6). This result is based on the anatomical location of the peak voxels in the activated clusters. The details of these regions are presented in Table 1.

### 3.2. Results of TEAS in the ST6 Group

Compared with the resting state, TEAS at the ST6 acupoint activated brain regions primarily in the bilateral middle temporal gyrus (BA21/37), bilateral postcentral gyrus (1/2/3/43), left cerebellum posterior lobe, left middle frontal gyrus (BA46), right precentral gyrus (BA4/6), left Inferior Parietal Lobule (BA5), and middle cingulate cortex (BA31). This result is based on the anatomical location of the peak voxels in the activated clusters. The details of these regions are presented in Table 2.

### 3.3. Results of TEAS in the Sham Point Group

Compared with the resting state, there was an area of slight brain activation in the bilateral temporal lobes and the left cerebellum in TEAS at the sham point. The details of these regions are presented in Table 3.

### 3.4. Comparison of the LI4 and Sham Point Groups

**Figure 3:**
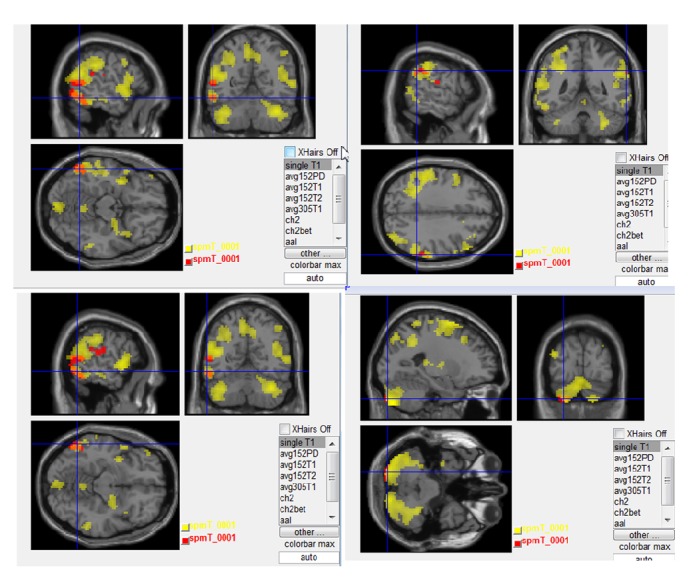
Comparison of brain regions activated between LI4 versus the sham acupoint. Yellow represents LI4 point activation. Red represents sham point activation. orange represent overlap regions. (*P* < 0.005, cluster >20 voxels, uncorrected).

Brain regions that were activated more by TEAS at LI4 than by TEAS at sham point were mostly located in the left middle temporal gyrus, right supramarginal gyrus, left inferior temporal gyrus, and left cerebellum posterior Lobe. The details of these regions are presented in Figure 3.

### 3.5. Comparison of the LI4 and ST6 Groups

**Figure 4:**
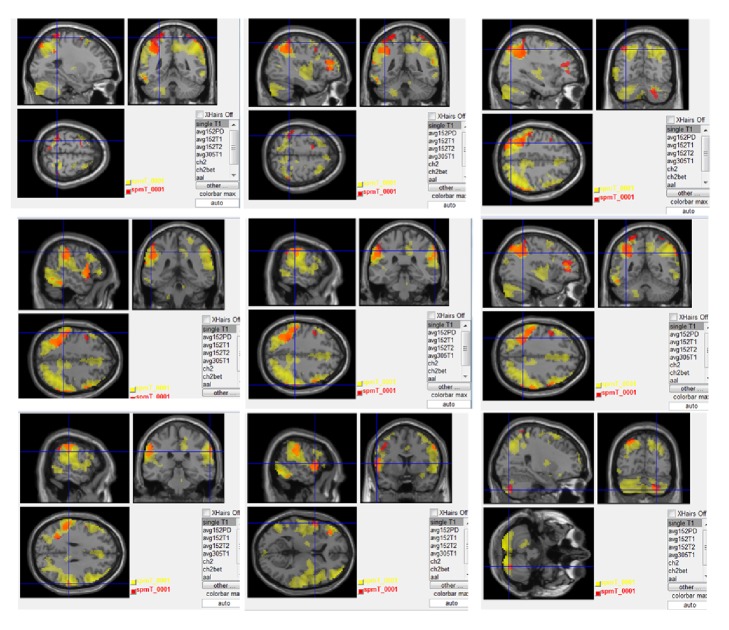
Comparison of brain regions activated between LI4 and ST6. red represent LI4 point activation. Yellow represents ST6 point activation. Orange represents overlap regions (*P* < 0.005, cluster >20 voxels, uncorrected).

In the same stimulus mode, the right LI4 showed the same activation area as the left ST6 acupoint: the left superior temporal gyrus, postcentral gyrus, middle frontal gyrus, inferior parietal lobule, inferior temporal gyrus, cerebellum posterior lobe, and right middle frontal gyrus. Brain regions that were activated of the right LI4 and the left ST6 were broadly overlapping and adjacent. The details of these regions are presented in Figure 4.

## 4. Discussion

The present study investigated the activation patterns of TEAS at LI4, ST6, and sham point, in subjects with Bell's palsy. The fMRI results showed extensive overlapping of activation areas between the LI4 and ST6 groups (Figure 4). Many common brain regions were activated, including the somatosensory cortex (e.g., postcentral gyrus, inferior parietal lobule), motor cortex (e.g., precentral gyrus), auditory cortex (e.g., superior temporal gyrus), prefrontal cortex (e.g., middle frontal gyrus), and the cerebellum. Some of these brain responses correlate with specific functional areas. The areas activated by sham point stimulation are not thought to be related to any specific needling location.

The postcentral gyrus belongs to the primary somatosensory area (SI) [18, 19]. Activation of this area has also been reported in two previous studies on the acupuncture at other acupoints [20, 21]. It is suggested that SI might be in part responsible for the effect of acupuncture [22].

The precentral gyrus is one of the main motor areas in the cerebral cortex that functions in association with other motor areas, such as the middle frontal gyrus, to plan and execute movements. The increased activated area in BA4 in this study corresponded to the somatotopic representation of the face primary motor cortex (M1), as supported by a previous study on Bell's palsy [23]. The left precentral gyrus (BA4) was activated by TEAS in this study; two other areas of increased activation were BA6 and BA10. BA6 is part of the dorsal premotor cortex, which plays an important role in the planning of complex, coordinated movements [24, 25], and BA10 is the most anterior lateral portion of the prefrontal cortex, which is activated by tasks that require integration of multiple relationships [26].

The superior temporal gyrus of the temporal lobe is the seat of the primary and secondary auditory cortex, which processes auditory information. Activation of the cerebellum may reveal that the cerebellar cortex can modulate different intracortical circuits within the contralateral primary motor cortex [27].

Most of the increased activations were located on the left side. The data were collected from patients with left-sided facial palsy with TEAS on right LI4 and left ST6.

Because TEAS is a specialized and complex stimulus of sensation, and the human brain is also a very complicated network in which various regions are closely and functionally connected [28], the TEAS stimulus could activate not only the somatosensory cortex but also many other regions functionally connected to the somatosensory cortex, such as the motor association cortex and cerebellum.

These findings support the view that neuronal responses to acupuncture observed with fMRI are inclined to be unique and specific [29]. Yan et al. reported that stimulation at LI4 could specifically elicit responses in the temporal pole [30]. Kong et al. indicated that LI4 acupuncture induced activation in the insula, superior parietal lobule, middle temporal gyrus, and postcentral gyrus [31].

Compared with previous experimental paradigms, we carried out, for the first time, correlative studies choosing two acupoints commonly used to treat Bell's palsy to explore the specific correlation between LI4 and the orofacial part. It is novel to use the method of TEAS at acupoints, making it possible to use fMRI to study facial acupoints.

This study had several limitations. First, we analyzed brain activation areas of only the task state and static state. Because acupuncture has a sustained effect, the next step is to analyze the activation areas in a post-TEAS resting state to explore the effects after acupuncture. Second, we selected only one sham point for the control group. As a next step, other acupoints adjacent to LI4 should be used as the control group, such results may be more credible. Third, the sample size in this experiment was small, so further studies with a greater number of subjects are warranted.

## 5. Conclusions

In conclusion, we revealed some features of neural responses to TEAS for patients with Bell's palsy. First, we found that TEAS at different acupoints and a sham point elicited different fMRI activation patterns in the human brain. This shows acupoint specificity. Furthermore, our findings suggest that LI4 and ST6 may elicit more specific and extensive activities in the human brain than the sham point. Second, brain regions that were activated with the right LI4 and the left ST6 were broadly overlapping and adjacent. These results may confirm the central mechanisms of the specific correlation between LI4 and the orofacial part. These results are helpful in interpreting the mechanism underlying the effect of acupuncture.

## Figures and Tables

**Table 1:** Activations for the group TEAS at LI4 point compared with a resting baseline are shown (*P* < 0.005, cluster >20 voxels, uncorrected).

Brain regions	MNI coordinates			BA	L/R	Voxels	*t* max
	*X*	*Y*	*Z*				
Superior temporal gyrus	−60	8	1	22	L	94	4.799
Precentral gyrus	−60	8	1	6	L	23	4.799
Cerebellum	−36	44	19		L	147	3.481
Middle frontal gyrus	−36	44	19	8	L	129	3.481
Inferior parietal lobule	63	−31	43	5	R	74	3.836
Postcentral gyrus	63	−31	43	1/2	R	52	3.836
Supramarginal gyrus	−27	−46	67	40	L	105	4.638
Postcentral gyrus	−27	−46	67	3	L	40	4.638
Superior parietal lobule	−27	−46	67		L	65	4.638
Inferior temporal gyrus	−57	−64	−11	20	L	121	4.293

**Table 2:** Activations for the group TEAS at ST6 point compared with a resting baseline are shown (*P* < 0.005, cluster >20 voxels, uncorrected).

Brain regions	MNI coordinates			BA	L/R	Voxels	*t* max
	*X*	*Y*	*Z*				
Middle temporal gyrus	−30	−64	49	21	L	245	7.570
Postcentral gyrus	−30	−64	49	1/2/3/43	L	258	7.570
Inferior parietal lobule	−30	−64	49	5	L	874	7.570
Middle frontal gyrus	−30	−64	49	46	L	284	7.570
Inferior frontal gyrus	39	59	−5	9	R	826	9.005
Middle cingulate cortex	39	59	−5	31	R	56	9.005
Postcentral gyrus	39	59	−5	1/2/3/43	R	338	9.005
Precentral gyrus	39	59	−5	7	R	202	9.005
Middle temporal gyrus	63	−64	11	37	R	123	4.795
Middle frontal gyrus	−24	17	61	31	L	94	4.256
Cerebellum posterior lobe	−15	−85	−47		L	907	7.627

**Table 3:** Activations for the group TEAS at sham point compared with a resting baseline are shown (*P* < 0.005, cluster >20 voxels, uncorrected).

Brain regions	MNI coordinates			BA	L/R	Voxels	*t* max
	*X*	*Y*	*Z*				
Supramarginal gyrus	−54	−67	−18	40	L	29	2.579
Superior temporal gyrus	−60	−28	16	41	L	42	3.877
Supramarginal gyrus	66	−34	31	22	R	21	3.382
Cerebellum	−51	−55	−14		L	22	4.358
